# Evaluation of the genetic diversity of six Chinese indigenous chickens

**DOI:** 10.5713/ajas.19.0606

**Published:** 2019-11-12

**Authors:** Yuzhu Sha, Caixia Gao, Meimei Liu, Shengguo Zhao

**Affiliations:** 1 College of Animal Science and Technology, Gansu Agricultural University, Lanzhou 730070, China; 2 State Key Laboratory of Veterinary Biotechnology, Harbin Veterinary Research Institute, Chinese Academy of Agricultural Sciences, Harbin 150069, China

**Keywords:** Genetic Diversity, Phylogeny, Indigenous Chicken, Maternal Origin

## Abstract

**Objective:**

The extensive breeding of commercial chickens has led to a sharp decrease in the resources of many indigenous chickens, especially the indigenous chickens in the southeastern coastal region, which are on the verge of extinction, and the indigenous chickens in the northwestern region of China, which are also at risk. However, there are few reports on the evaluation of genetic diversity and conservation of genetic resources of indigenous chickens in remote areas in the Northwest of China.

**Methods:**

In the present study, the genetic diversity and phylogenetic relationship of six indigenous chickens from different regions were studied based on variation in mitochondrial DNA control region (D-loop), and the degree of introgression from commercial breeds into these chickens was determined by the amount of haplotype sharing between indigenous and commercial breeds.

**Results:**

Twenty-five polymorphic sites and 25 haplotypes were detected in 206 individuals. Principal component analysis showed that the Jingning chicken had the highest genetic diversity among the six indigenous chickens. According to the degree of introgression, the six indigenous breeds may be involved in haplotype sharing with commercial breeds, and the introgression from commercial chickens into the Haidong chicken is the most serious.

**Conclusion:**

The genetic uniqueness of indigenous chickens has been eroded, so it is necessary to consider the protection of their genetic resources. Phylogenetic analysis suggests that the six indigenous chickens have two major matrilineal origins: one from Yunnan or its surrounding areas in China and the other from the Indian subcontinent.

## INTRODUCTION

Domestic animals have evolved genetic adaptations to a new environment, the farm, and have been subjected to strong human-driven selection, leading to remarkable phenotypic changes in morphology, physiology and behavior. Identifying the genetic changes underlying these developments provides new insight into the general mechanisms by which genetic variation shapes phenotypic diversity [1]. In the future, the genetic diversity of indigenous chickens will be needed to meet production demands in different environments, thereby achieving sustained genetic improvement and promoting rapid adaptation to changing breeding objectives [2,3]. The extensive breeding of commercial chickens has had an impact on resources of indigenous chicken breeds, especially indigenous chickens in the coastal areas of Southeast China, which are on the verge of extinction or even extinct, while the indigenous chickens in the inland northwestern region are only slightly affected but still face certain risks. However, the potential of indigenous breeds in some developing countries is often not fully documented and utilized. Therefore, it is necessary to protect the genetic resources of these indigenous chickens. This study will analyze the genetic diversity and phylogeny of six indigenous chickens in Northwest China based on sequences of the mtDNA D-loop, analyze the degree of introgression from commercial breeds to indigenous chickens, and finally quantify their genetic diversity and evaluate their conservation priorities.

## MATERIALS AND METHODS

### Ethics statement

All studies involving animal were carried out in accordance with the regulations for the Administration of Affairs Concerning Experimental Animal (Ministry of Science and Technology, China; revise in June 2004). Blood samples collection was approved by the ethics committee of Agricultural University. Sample collection were conducted according to the guidelines established by the Ethics Committee for the Care and Use of Laboratory Animals at Gansu Agricultural University.

### Sampling

Blood samples were collected on the Flinders Technology Associates cards (FTA) (Whatman, Inc., Buckinghamshire, UK) from six indigenous breeds (175 birds) of China: Jingning chicken (JN), Huining chicken (HN), Minqin chicken (MQ), Taiping chicken (TP), Beijing fatty chicken (BY), and Haidong chicken (HD) (Supplementary Table S1). Within each region, samples were collected from multiple households in different villages. To minimize the chances that the birds used from each village were related, a single bird was sampled from each household. In addition, we collected sequence information for 31 commercial chickens, including 13 Hy-line chickens in this study, and the mtDNA sequences of 18 other commercial chickens were obtained from NCBI (Supplementary Table S2). The study also used 11 sequences of Miao et al [4] and Liu et al [5] as reference sets (Supplementary Table S3).

### Polymerase chain reaction amplification and DNA sequencing

The D-loop region was amplified directly from the genomic DNA by polymerase chain reaction (PCR). The primer pair, L16750 (5′-AGGACTACGGCTTGAAAAGC-3′) and H547 (5′-ATGTGCCTGACCGAGGAACCAG-3′), described by Niu et al [6] was used to amplify the D-loop hypervariable region. In the primer names, L and H refer to the light and heavy chains, respectively, and the number designates the position of the 3′-end of the primer on the complete chicken mtDNA sequence [7]. PCR reactions were carried out in 30 μL volumes using 3.0 μL 10×PCR reaction buffer and MgCl_2_, 1 μL dNTP (2.5 mM), 1 μL of each primer, 0.3 μL Taq-polymerase, 2.0 μL Template DNA, and 21.7 μL ddH_2_O. The PCR cycle included the initial denaturation at 94°C for 10 min followed by 30 cycles of denaturation at 94°C for 30 s, annealing at 61°C for 30 s and extension at 72°C for 30 s with a final extension at 72°C for 10 min using GenAmp 9700 (Applied Biosystems, CA, USA). The PCR products were purified with Tiangen PCR purification kit (Tiangen, China) according to the manufacturer’s instructions. Sequencing reaction was performed by using Big Dye Terminator Cycle Sequencing Ready Reaction Kit (v3.1, Applied Biosystems, USA) and electrophoresis was done by a ABI 3130XL DNA Genetic Analyzer (Applied Biosystems, USA).

### Data analysis

The mtDNA nucleotide sequences obtained in this study were aligned by using the ClustalX program (http://www.igbmc.ustrasbg.fr/pub/ClustalX), and identical sequences were considered to be the same haplotype. The diversity parameters were estimated using DnaSP 5.0 [8]. Correlation analysis and principal component analysis (PCA) of the nucleotide diversity (Pi), the average number of nucleotide differences (K), and haplotype diversity (Hd) indices were performed [9]. Analysis of the degree of introgression from commercial breeds into indigenous breeds was performed in reference to the article of Zhang et al [10]. A haplotype median-joining network was constructed via Network 5.0 software to evaluate haplotype relationships [11]. A neighbor-joining tree was built using MEGA 7.0 software [12]. For convenience, the haplotypes were named according to the convention of Miao et al [4], in which (*) indicates a new haplotype.

## RESULTS

### Haplotype analysis

Analysis of the mtDNA D-loop sequences from the 206 samples showed a total of 25 nucleotide changes, which were grouped into 25 haplotypes (Table 1). The four largest haplotype groups were A01, A81*, E01, and E06. The A01 haplotype occurred at a frequency of 32.04% and was distributed in four indigenous breeds. The A81* and E06 haplotypes were distributed in only two indigenous breeds, and their haplotype frequencies were 7.77% and 13.59%, respectively. The E01 haplotype was distributed among the five indigenous breeds and the commercial breeds at a frequency of 12.14%, except HN chicken. The distribution of haplotypes and their frequencies are shown in Table 1. The HD chicken was the only indigenous breed with no unique haplotypes. Twenty-five polymorphic sites were found in the 397 bp sequenced, equaling an average of 6.30% polymorphic sites in the 206 samples. The nucleotide changes were characterized by transitions at 23 sites, transversions at 2 sites and no deletions or insertions.

### Genetic diversity analysis

The genetic diversity calculated for the six indigenous chickens and commercial chickens is shown in Table 3. The Pi ranged from 0.00353 to 0.01284, while the Hd was between 0.446± 0.105 and 0.928±0.034. Among the breeds, the Pi and Hd of the JN chicken were the highest, at 0.01284 and 0.928±0.034, respectively. The number of haplotypes, the number of unique haplotypes and the K in the JN chicken were also the highest among the six indigenous chickens. The lowest value of Pi was observed in the BY chicken (0.00353), and the lowest Hd was observed in the HN chicken (0.446±0.105).

To comprehensively evaluate the genetic diversity of the indigenous chickens, correlation analysis and PCA were performed on Pi, K, and Hd (Tables 2, 3). Correlation analysis showed that Pi, K, and Hd were positively correlated with each other. As shown in Table 2, the correlation coefficient between Pi and K was 1.000, which was higher than the other two, indicating that the Pi and the K were strongly positively correlated. The PCA results are shown in Table 3. We derived a composite principal component value (Fz), the magnitude of which comprehensively reflects the level of genetic diversity, and found the JN chicken to have the highest genetic diversity. In addition, we also found that commercial chickens have higher genetic diversity than HN, TP, and BY indigenous chickens.

### Analysis of introgression from commercial breeds into six indigenous breeds

Twenty-five haplotypes were identified in 206 individuals of the indigenous and commercial breeds. The commercial breeds were distributed among 6 haplotypes. The haplotypes of commercial breeds were shared with all indigenous breeds except for the HN chicken. As shown in Table 4, the ratio of the number of indigenous chickens sharing the haplotype to the total number of indigenous chickens (Sc/S) indicates the degree of introgression from commercial breeds to indigenous breeds. In this study, the Sc/S ratios of all the indigenous breeds except the HN chicken ranged from 4.76% (TP) to 50.00% (HD), with the HD chicken exhibiting the highest degree of introgression.

### Phylogenetic and Median-joining network analysis

**Figure 1:**
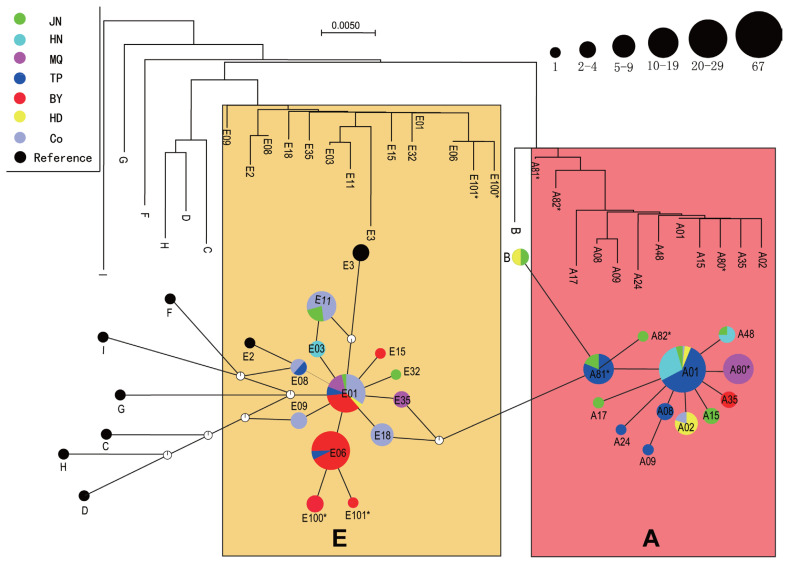
Neighbor-joining phylogenetic tree of the six breeds and median network profile of the mtDNA D-loop haplotypes. The circle size corresponds to haplotype frequency. The small circles with white and black edges are false points, representing the mutation events that should theoretically occur but were not found in this analysis. E, E haplogroup that originated on the Indian subcontinent; A, A haplogroup that originated in Yunnan or surrounding areas.

Phylogenetic trees were constructed based on nucleotide sequences from the six indigenous breeds, and median-joining network profiles were constructed based on 25 haplotypes and 11 reference sequences (Figure 1). As shown in the figure, phylogenetic trees clustered haplotype A into one cluster and haplotype E into another cluster, and the median-joining network profiles corresponded to the phylogenetic tree. We combined the phylogenetic tree with the median-joining network profiles and found that the 25 haplotypes composed of six indigenous chickens and commercial chickens were mainly divided into two haplogroups (A and E), while the eight other haplotypes (F, H, I, G, C, D, E2, and E3) that were independent of these two haplogroups in the studies of Miao et al [4] and Liu et al [5] were not observed in any of the six indigenous chickens. In addition, we found haplotype B, which was independent of haplogroups A and E, in only JN and HD chickens. Only one false point was found between haplogroups A and E, which were far away from each other, while no false points were found between haplogroups B and A, which were also far away from each other. The size of the circles in the median-joining networks represents the corresponding haplotype frequency, with the haplotype frequencies of A01, A81*, E01, E06, and E11 being the largest. In haplogroup A, some haplotypes of class A with low frequencies take the dominant haplotype A01 with high frequencies as the center and present a star-shaped structure, while in haplogroup E, haplotype E01 is taken as the center and presents a star-shaped structure. We also found that haplotype E06, which is similar to haplotype E01 in frequency, is an independent center that gives rise to two new haplotypes (E100* and E101*). Such a star-shaped structure indicates that the haplotype of each satellite is probably the haplotype of the maternal ancestor, with the dominant haplotype at the center, and the existing diversity in the haplotype sequence is formed through more or less base variation. Therefore, the two haplogroups found in this study may have different maternal ancestral haplotypes. Twelve and 10 haplotypes were assigned to haplogroups A and E, respectively, in the six indigenous breeds. TP, HD, and HN chickens are mainly distributed in haplogroup A, BY, and commercial chickens are mainly distributed in haplogroup E, and JN and MQ chickens are distributed in both haplogroups A and E.

## DISCUSSION

### Genetic diversity of six indigenous chickens

The Hd, Pi, and K reflect different aspects of genetic diversity, while PCA can comprehensively evaluate genetic diversity. PCA is a statistical process that reduces the dimensionality of a data set by transforming the data into a new set of variables [9]. Therefore, PCA was used to analyze the diversity parameters Hd, Pi, and K, and a comprehensive principal component value, Fz, was obtained to reflect the genetic diversity (Table 3). Fz was between −0.1643 and 2.800, with the JN chicken exhibiting the highest value, followed by the HD chicken and the MQ chicken, indicating that the JN chicken has the most genetic diversity. In addition, we found a special case in which commercial chickens have higher genetic diversity than HN, TP, and BY chickens, which may be related to the breeding process of commercial chickens. Commercial chicken breeding generally involves haplotypes with many indigenous chickens as the original material and intensive production, so commercial chickens have a higher genetic diversity than some indigenous chickens.

The genetic diversity of the six indigenous chickens is shown in Table 3. The Pi index was between 0.00353 and 0.01284, and the Hd index was between 0.446 and 0.928. The values of these two indices are lower than those calculated by Silva et al [13] for indigenous chicken populations in Sri Lanka. The differences in the diversity indices between the six indigenous breeds and the Sri Lankan indigenous chickens can be explained by Sri Lanka being located in the southeastern part of the Indian subcontinent, which was an important center of origin for indigenous chickens [5,14,15]. However, the Pi indices observed in this study were higher than those estimated by Liu et al [5] for clades of chickens from Europe, the Middle East, Southeast Asia and East Asia and by Oka et al [16] for Japanese native chickens. Higher genetic diversity in chickens is indicative of the center of origin of the species and confluence, where genetic variation has been generated and accumulated over long periods of time [17]. Japanese native chickens are believed to have been established from native chicken populations from other East and Southeast Asian countries, which accounts for the lower genetic diversity in the foundation populations of Japanese native chickens than in the original populations in other Asian countries [16]. Liu et al [5] believe that Yunnan or its surrounding areas is the center of origin for the domestic chicken, and the geographical distribution of the six Chinese indigenous chickens examined in this study is closer to Yunnan, China [5]. This difference in geography could explain the higher genetic diversity of the six Chinese indigenous chickens observed in this study, especially the JN chicken, which had the highest level of genetic diversity. This high level of diversity may be because JN chickens are distributed in the mountainous areas of northwestern China, where they range freely with little strict manual breeding. Due to geographical isolation, there is no hybridization with the introduced breeds, so the diversity is the most abundant. The genetic diversity of TP and BY chickens was the lowest, and TP chickens were distributed in northwestern China, where the abundance of chickens, degree of artificial breeding, and gene flow are lower, resulting in lower diversity.

### Introgression from commercial breeds into six indigenous breeds

Due to the large-scale production and promotion of commercial breeds, the indigenous chicken breeds with relatively low egg and meat production are threatened, and the amount of breeding is gradually reduced or even eliminated. Such commercial breeding reduces the genetic diversity of indigenous chicken breeds [18], and the extensive breeding of commercial chicken breeds also has an impact on indigenous chicken breeds in remote areas. In this study, thirty-one individuals from commercial breeds were distributed among six haplotypes, and the unique haplotypes E09 and E18 indicate that commercial breeds have more breeding backgrounds. Four haplotypes are shared among the six indigenous breeds, suggesting that these six indigenous chickens are involved in commercial breeding or that these commercial breeds have been crossed with the indigenous chickens; regardless, because of the large number of commercial chickens, the number of individuals sharing these haplotypes is likely to be relatively large. A total of 50% of the HD chickens share a common haplotype with commercial chickens. Therefore, in the protection of HD chickens, it is necessary to identify individuals that do not share haplotypes with commercial chickens and protect those individuals as much as possible. Other breeds have relatively few individuals that share a haplotype with commercial breeds, and no traces of commercial chickens are found in the HN breeds, so full population protection should be considered.

### Maternal origins of six indigenous chickens

According to Ohno [19], half-sisters should inherit the same mitochondrial genome from their mothers, but within a certain period of time, their offspring can also establish an independent lineage due to the accumulation of mutations. Since the Darwinian era, the origin and domestication of domestic chickens have attracted attention in many disciplines. Many studies have been carried out on the maternal origin of domestic chickens using mtDNA sequences. These studies indicate that red jungle fowl are the original ancestors of domestic chickens [20]. However, several independent domestication events occurred in southern China, South Asia, and Southeast Asia, possibly involving multiple maternal origins [5,21]. Liu et al [5] analyzed mtDNA D-loop hypervariable regions in 834 chickens in Eurasia and 66 red jungle fowl in China and Southeast Asia and concluded that chickens originated on the Indian subcontinent and in southwestern China and its surrounding areas [5]. Niu et al [6] studied the phylogenetic relationships of six indigenous chickens in China and showed that the chickens descended from red jungle fowl in Thailand and its surrounding areas. Song et al [22] reported that six native Chinese chickens closely related to *G. g. gallus*, *G. g. jabouillei*, and *G. g. spadiceus* distributed in Yunnan Province and Laos may have originated from red jungle fowl subspecies in Yunnan, Laos, and Vietnam and its surrounding areas. Gong et al [23] reported that Piao chickens have five maternal origins. In this study, the phylogenetic trees of the six indigenous chickens were mainly divided into two clusters (A and E), suggesting two major maternal origins (Figure 1). The A and E haplogroups in this study correspond to the A and E haplogroups in the study of Liu et al [5]. Haplogroups A and B are distributed throughout the world (except Africa), having originated in Yunnan or its surrounding areas, and haplogroup E is also widely distributed in the maternal pedigree, mainly having originating on the Indian subcontinent [4,5]. Based on these patterns, TP, HN, and HD chickens, which clustered into the A haplogroup, likely originated in Yunnan or surrounding areas, and BY chickens and commercial chickens, in the E haplogroup, mainly originated on the Indian subcontinent. JN and MQ chickens may have been distributed in both the A and E haplogroups due to gene flow.

## CONCLUSION

Our results reveal high genetic diversity among the six indigenous chickens, especially the JN chicken, which may be related to their distribution in remote rural areas. Therefore, it is necessary to consider the protection of their genetic resources. According to the degree of introgression from commercial breeds into indigenous chickens, the six indigenous breeds may be involved in commercial breeding, and the introgression from commercial chickens into HD chickens is the most serious. In contrast, no traces of commercial chickens were found in the HN chicken, so full population protection should be considered. Phylogenetic analysis suggested that these six indigenous chickens have two major maternal origins: one from the Yunnan region of China or surrounding areas and the other from the Indian subcontinent.

## Figures and Tables

**Table 1:** Polymorphic sites and distribution of haplotypes

Haplotypes	Variable sites	Frequency							
	
	2811122222222222223333333133690111223334456113479937970272578936610502216	JN1)	HN1)	MQ1)	TP1)	BY1)	HD1)	Co1)	Total
B	CATTTACATACAGATTTCCTCAACT	2					2		4
A01	...C...G..T....C...C.....	3	19		41		3		66
A15	...C...G..T....C...C....C	2							2
A80*	...C...G..TG...C...C.....			10					10
A35	...C...G..T..G.C...C.....					2			2
A02	...C..TG..T....C...C.....						4	1	5
A48	...C...G..T....C...C.G...	1	3						4
A08	...C.G.G..T....C...C.....				2				2
A09	...C.G.G..T........C.....				1				1
A17	...CC..G..T....C...C..G..	1							1
A24	..CC...G..T....C.........				1				1
A81*	.......G.......C...C.....	3			13				16
A82*	.......G.......C...C..G..	1							1
E35	.......GC.....CCCT.C.....			3					3
E01	.......GC.....CCCTTC.....	1		4	2	8	1	9	25
E32	.......GC...A.CCCTTC.....	1							1
E15	.......GC.....CCCTTC...A.					1			1
E11	.......GCG....CCCTTCT....	3						10	13
E03	.......GC.....CCCTTCT....		4						4
E06	....C..GC.....CCCTTC.....				2	26			28
E100*	.G..C..GC.....CCCTTC.....					3			3
E101*	G...C..GC.....CCCTTC.....					1			1
E08	.......GC.....C.CTTC.....				1			1	2
E09	.......GC.....CCCTTC.G...							3	3
E18	.......GC......CCTTC.....							7	7
Total		18	26	17	63	41	10	31	206

Numbers indicate nucleotide base position in mitochondrial D-loop region; frequency represents the number of individuals per haplotype; dot (.) represents the identical nucleotide with the haplotype B.

1) JN, Jingning chicken; HN, Huining chicken; MQ, Minqin chicken; TP, Taiping chicken; BY, Beijing fatty chicken; HD, Haidong chicken; Co, Commercial chicken.

**Table 2:** Correlation matrix between indexes

Item	Pi	K	Hd
Pi	1.000	1.000	0.645
K	1.000	1.000	0.645
Hd	0.645	0.645	1.000

Pi, nucleotide diversity; K, average number of nucleotide differences; Hd, haplotype (gene) diversity.

**Table 3:** Diversity parameters of indigenous and commercial chickens

Breed1)	N	S	Hn	Hu	Pi	K	Hd±SD	Fz (F1)	Rank
JN	18	17	10	4	0.01284	5.098	0.928±0.034	2.800	1
HD	10	11	4	0	0.00890	3.533	0.778±0.091	0.931	2
MQ	17	8	3	2	0.01004	3.985	0.603±0.098	0.813	3
Co	31	12	6	2	0.00503	1.996	0.774±0.037	−0.474	4
HN	26	9	3	1	0.00599	2.378	0.446±0.105	−1.116	5
TP	63	12	8	3	0.00467	1.853	0.539±0.064	−1.311	6
BY	41	12	6	4	0.00353	1.400	0.565±0.078	−1.643	7

N, the size of populations; S, number of variable sites; Hn, the number of haplotypes; Hu, the number of unique haplotypes; Pi, nucleotide diversity; K, average number of nucleotide differences; Hd, haplotype (gene) diversity; SD, standard deviation; Fz (F1), composite principal component value.

1) JN, Jingning chicken; HD, Haidong chicken; MQ, Minqin chicken; Co, Commercial chicken; HN, Huining chicken; TP, Taiping chicken; BY, Beijing fatty chicken.

**Table 4:** Analysis of indigenous chicken haplotypes shared with commercial chickens

Breed1)	Sc	S	Sc/S (%)
JN	4	18	22.22
HN	0	26	0.00
MQ	4	17	23.53
TP	3	63	4.76
BY	8	41	19.51
HD	5	10	50.00

Sc, indicates the number of individual chickens sharing a haplotype with commercial chickens; S, The total number of individuals representing indigenous chickens.

1) JN, Jingning chicken; HN, Huining chicken; MQ, Minqin chicken; TP, Taiping chicken; BY, Beijing fatty chicken; HD, Haidong chicken.
