# Supplementary material for: Evaluation of the genetic diversity of six Chinese indigenous chickens
Source: Asian-Australas J Anim Sci. 2019 Nov 12;33(10):1566–72. doi: 10.5713/ajas.19.0606 (PMC7463083; doi:10.5713/ajas.19.0606)
Supplement: Supplementary file 1 [file ajas-19-0606-suppl.pdf]

Table S1 Sample information

Breeds	ID	Size	Type	Breeds/Populations	Resource	Location
Jingning	JN	18	Indigenous	Jingning	This study	Jingning, Gansu, China
Huining	HN	26	Indigenous	Huining	This study	Huining, Gansu, China
Minqin	MQ	17	Indigenous	Minqin	This study	Minqin, Gansu, China
Taiping	TP	63	Indigenous	Taiping	This study	Kang, Gansu, China
Beijing fatty chicken	BY	41	Indigenous	Fatty chicken	This study	Beijing, China
Haidong	HD	10	Indigenous	Haidong	This study	Haidong, Qinghai, China
Commeicial	CO	13	Commercial breeds	Hy-line	This study	Lanzhou, Gansu, China
Commeicial	CO	18	Commercial breeds	Other commercial breeds	NCBI	

Table S2 Sequence information from 18 commercial chickens in NCBI

Type	Breeds/Common Name	Accession No.
Commercial	Barred Plymouth Rock	AB007719
Commercial	White Leghorn	AB007723
Commercial	White Leghorn	AB268508
Commercial	Rhode Island	AB268513
Commercial	White Leghorn	AB268521
Commercial	White Leghorn	AF128325
Commercial	White Leghorn	AF128326
Commercial	White Leghorn	AF128327
Commercial	White Leghorn	AF128328
Commercial	White Leghorn	AF128329
Commercial	White Leghorn	AP003317
Commercial	Barred Plymouth Rock	AP003318
Commercial	White Leghorn	AP003580
Commercial	New Hampshire Red	AY235570
Commercial	New Hampshire Red	AY235571
Commercial	Barred Plymouth Rock	D82920
Commercial	White Leghorn	D82923
Commercial	White Leghorn	D82925

Table S3 Reference sequence information

Breeds/Common Name	Type	Accession No.	Location	Haplogroup	Haplotype	Reference
Nixi	Indigenous	AF512253	China: Yunnan	A	A01	Liu et al. 2006 Mol Phylogenet Evol
commercial broiler dam line A (BRD_A)	Commercial	AM746036	Japan	B	B01	Muchadeyi et al. 2008 Anim Genet
HubeiSilky	Indigenous	AF512197	East Asia	C1	C01	Liu et al. 2006 Mol Phylogenet Evol
Malawi Village Chicken	Indigenous	AM746024	Malawi	D	D46	Muchadeyi et al. 2008 Anim Genet
	Indigenous	AY644998	Europe	E	E01	Liu et al. 2006 Mol Phylogenet Evol
	Indigenous	GU448386	Northeast India	E2	E17	Miao et al. 2013 Heredity
	Indigenous	EU847810	Northern India: Haryana	E3	E18	Kanginakudru et al. 2008 BMC Evol Biol
Lv'erwu	Indigenous	AY392249	China: Yunnan	F	F01	Liu et al. 2006 Mol Phylogenet Evol
Lv'erwu	Indigenous	AY392246	China: Yunnan	G	G01	Liu et al. 2006 Mol Phylogenet Evol
Lijiang	Indigenous	GU447838	China: Yunnan	H	H01	Miao et al. 2013 Heredity
	Indigenous	GU448422	Northeast India	I		Miao et al. 2013 Heredity
